# Improving Perinatal Care in the Rural Regions Worldwide by Wireless Enabled Antepartum Fetal Monitoring: A Demonstration Project

**DOI:** 10.1155/2015/794180

**Published:** 2015-01-26

**Authors:** Roberto Tapia-Conyer, Shelley Lyford, Rodrigo Saucedo, Michael Casale, Hector Gallardo, Karen Becerra, Jonathan Mack, Ricardo Mujica, Daniel Estrada, Antonio Sanchez, Ramon Sabido, Carlos Meier, Joseph Smith

**Affiliations:** ^1^Instituto Carlos Slim de la Salud, Insurgentes Sur 3500, 14060 Ciudad de México, DF, Mexico; ^2^Gary and Mary West Health Institute, 10350 N Torrey Pines Road, La Jolla, CA 92037, USA; ^3^Servicios de Salud de Yucatán, Calle 72, No. 463 Entre 53 y 55, Centro, 97000 Merida, YUC, Mexico

## Abstract

*Background*. Fetal and neonatal morbidity and mortality are significant problems in developing countries; remote maternal-fetal monitoring offers promise in addressing this challenge. The Gary and Mary West Health Institute and the Instituto Carlos Slim de la Salud conducted a demonstration project of wirelessly enabled antepartum maternal-fetal monitoring in the state of Yucatán, Mexico, to assess whether there were any fundamental barriers preventing deployment and use. *Methods*. Following informed consent, high-risk pregnant women at 27–29 weeks of gestation at the Chemax primary clinic participated in remote maternal-fetal monitoring. Study participants were randomized to receive either prototype wireless monitoring or standard-of-care. Feasibility was evaluated by assessing technical aspects of performance, adherence to monitoring appointments, and response to recommendations. *Results*. Data were collected from 153 high-risk pregnant indigenous Mayan women receiving either remote monitoring (*n* = 74) or usual standard-of-care (*n* = 79). Remote monitoring resulted in markedly increased adherence (94.3% versus 45.1%). Health outcomes were not statistically different in the two groups. *Conclusions*. Remote maternal-fetal monitoring is feasible in resource-constrained environments and can improve maternal compliance for monitoring sessions. Improvement in maternal-fetal health outcomes requires integration of such technology into sociocultural context and addressing logistical challenges of access to appropriate emergency services.

## 1. Introduction

While most historical efforts to improve health outcomes in rural communities across the world have focused on prevention and treatment of infectious disease, in recent years attention has turned to noncommunicable disease, as well as maternal-child health (MCH). Within Mexico, the nationwide strategy* Equal Start in Life* was implemented in 2001 to enable pregnant women and families to access quality health services [1]. The* Equal Start in Life* program is focused on achieving universal coverage and equal quality care conditions to women during pregnancy, childbirth, and postpartum, as well as to children (boys and girls) from birth through two years of age. This program, which included mobile health units, increased roles of community healthcare workers (CHWs), enhanced family planning services, and made significant advances in regard to maternal and child mortality. In 2007, the Instituto Carlos Slim de la Salud (ICSS), part of the Carlos Slim Foundation, which was established to create sustainable health initiatives for Mexico and Latin America, developed the AMANECE model to improve MCH outcomes [2]. Like the* Equal Start in Life* program, the AMANECE model focuses on maternal and newborn care, and that operates in joint partnership with the federal government, state government public health facilities, and the private sector. Conceptually, AMANECE aims to improve MCH outcomes by creating networks of care that range from community-level health services to highly specialized hospitals; using technology to enhance clinical communication and decision making; and focusing on prevention and early detection of pregnancies, systematic assessment of health risks during prenatal care, and timely treatment of signs of alarm to either prevent the incidence or reduce the severity of obstetric complications. AMANECE strengthens these networks of care by deploying a number of innovations, including a traditional midwife kit containing low-cost medical instruments; AMANECEnet, a tablet-based program to help CHWs identify pregnant women and assess them in the community; SI-VA Amanece, a hybrid online-offline risk assessment platform to ensure high-quality prenatal care at the clinic; cloud software to facilitate referral to regional hospitals; and a hospital rapid response team to deploy a standardized obstetric emergency protocol. To date, the AMANECE program has made significant advances in MCH within Mexico and Latin America. With the aim of furthering achievement of the Millennium Development Goals, ICSS has partnered with the Ministry of Health of Mexico to create networks of care in 15 states in highly populated urban areas and marginalized rural communities; in addition, it has partnered with the Bill and Melinda Gates Foundation and the Government of Spain to implement MCH interventions in Central America.

**Figure 1:**
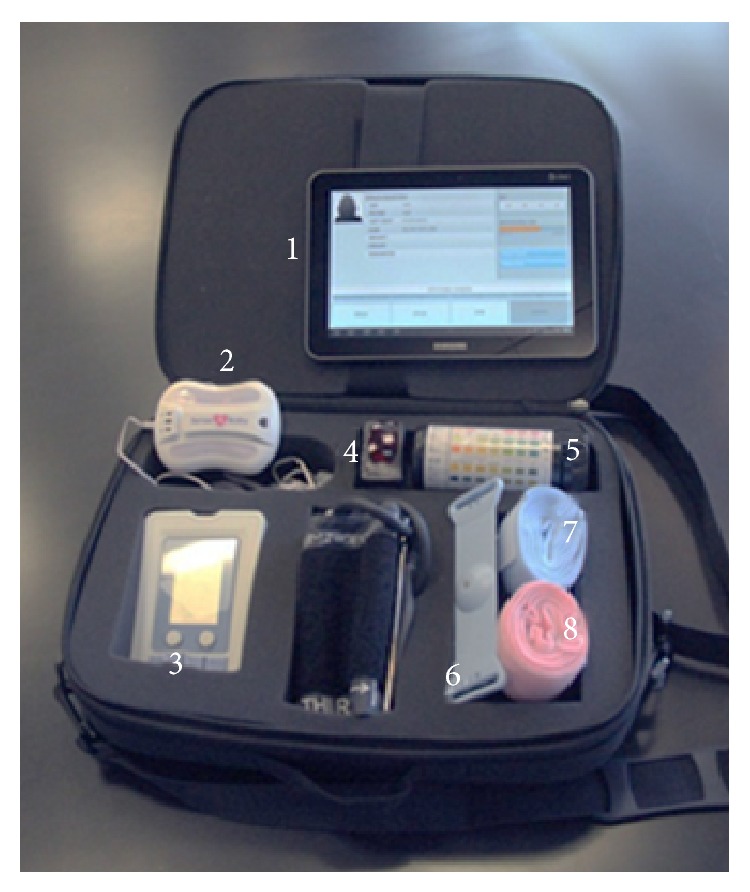
MiBebe fetal remote monitoring kit prototype technology with components as deployed in YUC, Mexico: (1) tablet/phone with fetal monitoring app; (2) fetal monitoring central unit; (3) FORA (blood pressure and glucometer); (4) pulse oximeter; (5) urine strips; (6) Toco; (7) elastic strap for central unit; (8) elastic strap for Toco.

In 2010 the ICSS and the Gary and Mary West Health Institute (WHI), a nonprofit medical research organization based in La Jolla, CA, collaborated to conduct a feasibility demonstration project of remote, wireless antepartum fetal monitoring for high-risk pregnant women. WHI was established to help bring forth solutions to increase access to, improve the quality of, and lower the cost of healthcare. For this deployment, WHI contributed the MiBebe fetal remote monitoring prototype kits (Figure 1) to a rural clinic in Chemax, YUC, which is part of the AMANECE network. The project, called Salud Maya, was established within the AMANECE network of Valladolid, YUC, México. With the deployment of Salud Maya, WHI and ICSS introduced a clinic-based electronic maternal-fetal monitoring prototype kit to improve broad clinical education and communication to and from Valladolid General Hospital.

Currently, prenatal care in the AMANECE network of care consists of paper-based medical records and a standard set of tests (urine markers, blood pressure, blood glucose, ultrasound testing, and select vaccinations). Typically, “low-risk” women are provided four prenatal visits (12, 20, 28, and 35 weeks of gestation) in a primary care clinic, after which they are referred to Valladolid General Hospital for near-term care and delivery. If the primary care physician (PCP) determines high-risk status during prenatal care, these women are referred to Valladolid, where an OB/GYN physician determines how frequently patients should come in for prenatal visits. The average distances women must travel to the Chemax clinic and Valladolid General Hospital are 1.2 km and 30.4 km, respectively. Due to the distance and cost of travel to Valladolid, many high-risk women do not comply with referrals to the hospital. Given the known benefits of fetal monitoring [3, 4], it is expected that providing pregnant women with increased access to monitoring will likely result in increased adherence to monitoring, thereby improving healthcare outcomes.

The present investigation focused on better understanding the feasibility of implementing a remote wireless antepartum fetal monitor prototype kit within rural Mexico through the Salud Maya project. We specifically addressed technical, infrastructural, and sociocultural challenges. It is important to note that, in performing this feasibility study, we also sought to address existing cultural, economic, and structural barriers to fetal monitoring.

## 2. Methods

### 2.1. Setting

The Salud Maya project was implemented in the jurisdiction of Valladolid, which encompasses the city of Valladolid and surrounding indigenous communities such as Chemax. Although most of the population speaks Spanish in the Chemax community, approximately 37% speak Mayan. The average yearly income in Chemax is less than US $1,000.00, and most of the population has an education at or below the eighth grade. Valladolid General Hospital, located in the city of Valladolid, is a 40-bed hospital that serves as a referral center from 5 urban and 13 rural clinics. As one of the referring rural clinics, the Chemax clinic serves the population southeast of Valladolid. The Chemax clinic is a primary care facility of ~180 square meters, containing four examination rooms staffed by a medical director, four PCPs, and eight community nurses. Prior to the project, the clinic had no phone or internet access. The standard care for fetal monitoring included a fetoscope and an obstetric tape measure. The clinic also had a standard supply of nutritional packages for pregnant women.

### 2.2. Project Phases

The Salud Maya project consisted of two phases. Phase I included importing prototype kits into Mexico, setting up clinics where the AMANECE network had been created to deploy the project, performing on-site demonstration of prototype technology kit use, training and proficiency certification in maternal-fetal monitoring, establishing connectivity and electric power, and creating a fetal monitoring room within each clinic. We granted final study initiation certification to the clinics only after each trainee, under supervision of the MD field researcher, had passed the technical certification. The certification test required trainees to demonstrate an understanding of the system and proficiency in using it on actual participants. Test data were reviewed by a participating OB/GYN specialist, and clinic certification was granted only after each trainee demonstrated proficiency during at least ten consecutive monitoring sessions and a final independent submission was completed.

Phase II (Figure 3) included recruiting subjects and deploying the fetal monitoring intervention. To create social awareness and improve recruitment efforts, the project was introduced to the public health and medical community in Valladolid General Hospital, to the Chemax clinic, and to the state authorities. In addition, to increase awareness of the program, a vehicle driven around the Chemax community delivered a series of health announcements via a public announcement system. The clinic supported these efforts to raise awareness by conducting monthly pregnancy health fairs and support groups, both of which gave potential participant women the opportunity to talk with peers and traditional midwives. Participants at 27–29 weeks of pregnancy with evidence of one of the high-risk inclusion criteria and the absence of exclusion criteria (Table 1) were enrolled after providing a written informed consent. At the first appointment, all potential participants were encouraged to bring a family member who wished to attend and witness the informed consent process.

### 2.3. Subject Description

Recruitment commenced on September 5, 2012, and ended on July 30, 2013, with a total of 172 enrolled women. Nineteen were excluded because they did not meet the inclusion criteria for maternal and gestational age, an obstetric event/delivery was not recorded, or they attended less than two visits. Following exclusion, there remained 153 eligible women who were randomly assigned to either the Salud Maya intervention (*n* = 74) or the standard care control group (*n* = 79).  Table 2 provides relevant study subject demographic information. No demographic characteristics were significantly different between the two groups (*P* > 0.05).

Two Mayan-speaking nurses were selected as part of the Chemax clinical team to assist with clinical data collection and address the language barriers between physicians and patients. Patient communications were mainly provided in Mayan.

Women enrolled in the study group attended their scheduled visits at the Chemax primary clinic. During each fetal monitoring visit, nurses collected urine samples and measured height, weight, blood pressure, and blood glucose; the PCP measured fundal height and reviewed and assessed clinical data; nurses performed fetal monitoring for 30 minutes, after which all data were uploaded to the cloud, reviewed at the hospital, and recorded as reactive or nonreactive. Clinical management was determined by the hospital OB/GYN specialist. Participants in the study group typically received fetal monitoring at 1- to 2-week intervals. Women enrolled in the control group and who served as the baseline cohort were referred for fetal monitoring to Valladolid General Hospital where they received standard care.

### 2.4. Study Materials

**Figure 2:**
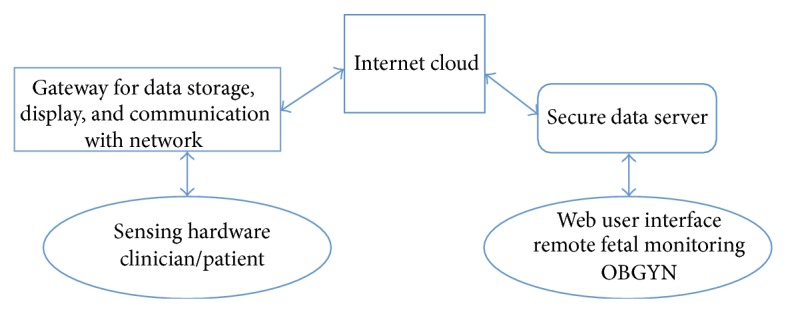
Remote maternal-fetal monitoring. Information is collected from the sensor hardware and uploaded to a secure cloud-based storage via Bluetooth. Data can then be accessed remotely by a clinician in a remote location.

**Figure 3:**
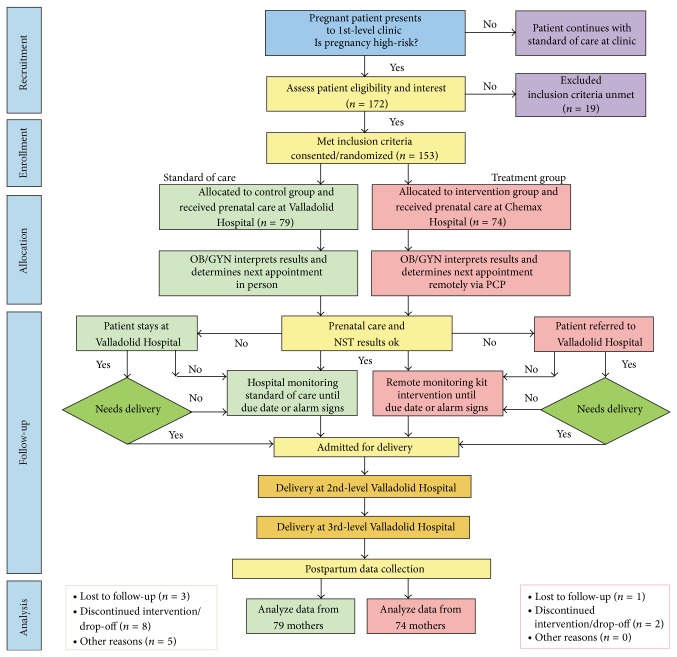
Flow chart illustrating the intervention process for participants.

The core of the WHI technology kits is the MiBebe fetal remote monitor prototype, which includes a fetal ultrasound heart monitor and a uterine tocodynamometer. The kit also contains tools to capture maternal blood pressure, blood glucose, and urinary protein values (Figure 1). Through a Bluetooth interface and Web access, the information is securely stored in the cloud and is available for reading at any hospital or remote location (Figure 2). Additional technical information on the prototype system and its architecture can be found in a separate publication [5].

### 2.5. Study Outcomes

The primary outcome of interest was adherence to antepartum clinic visits. To understand this, we compared the adherence rate for participants enrolled in the demonstration project to that of the baseline cohort.

In order to assess cultural/structural barriers that may impact program adoption, a follow-up questionnaire was established. The pregnant women, their husbands/partners, and traditional midwives as well as select health personnel who were in contact with the remote monitoring prototype kits were randomly sampled and queried regarding acceptability and satisfaction with the program. An independent bilingual team (Universidad de Oriente, Valladolid, YUC) conducted the interviews in either Spanish or Mayan (Tables 3, 4, and 5). Interviewers administered the questionnaire in open-ended face-to-face interviews conducted at the participant's home and video recorded.

In addition, during the project, the engineers and clinicians at WHI were responsive to all hardware and software potential errors via a bidirectional study feedback form. Potential errors were noted and recorded by individuals at the clinics via computer software spreadsheets that were stored locally and subsequently shared electronically with the team at WHI. The reported errors were classified as one of the following: (1) hardware, (2) software, (3) connectivity (cellular or webpage), or (4) other. Data errors were reviewed again later to assess the feasibility of the project.

Finally, clinical data were collected (1) upon enrollment, (2) during the monitoring sessions, and (3) after delivery. All clinical data collected are listed in Table 6. Economic impact questionnaires were independently administered to 30 pregnant mothers and their husbands/partners (data reported separately).

Since the primary aim of the present paper was to determine the overall feasibility of implementing the novel remote fetal monitoring system, we examined the results of each data type mentioned above to assess whether there were any fundamental barriers preventing the implementation of the Salud Maya demonstration project.

## 3. Results

### 3.1. Maternal and Infant Outcomes

Data analysis indicated no differences between individuals receiving the intervention and the control with respect to the proportion of deliveries at home versus hospital or in terms of term gestation, birth weights, or obstetric complications due to preeclampsia, eclampsia, hemorrhage, or sepsis (Table 7).

Adherence, the major outcome measure, was defined as attending scheduled fetal monitoring appointments, either through monitoring on-site at Chemax for the study group or through patient's referral to Valladolid Hospital for antepartum testing for the baseline cohort. Participants in the study group attended 481 of the 510 scheduled fetal monitoring appointments at Chemax, resulting in a compliance rate of 94.3%. Of remotely monitored participants, 40 study participants were referred to the hospital, based on observations from their remote monitoring, but only 17 complied, resulting in a referral compliance rate of 42.5%.

For the control group, the actual number of attended fetal monitoring appointments was 111. Out of a potential 246 recommended visits for antepartum monitoring to Valladolid General Hospital, this represents an approximate 45% adherence rate.

### 3.2. Interviews

Qualitative analysis of the interviews was performed to identify potential sociocultural barriers related to acceptance and adoption of the novel fetal monitoring kit demonstration project. Transcripts of the interviews conducted in Mayan were reviewed and the overarching themes extracted and analyzed. Among the pregnant women, the main perceived benefit of the program was the ability to hear the baby's heart, as they could infer the baby's health. Mayan women are generally very shy and reluctant to talk about themselves, so the fact that interviews were performed by an independent team may have limited the responses. Nonetheless, the Mayan pregnant women stated they were comfortable having a nurse perform the fetal monitoring session. Among the spouses/partners, the most common perception was the benefit of monitoring for the health of the baby. That they perceived the remote monitoring kit to be a positive intervention primarily in regard to infant health is not surprising, as Mayan indigenous communities have strong ties to the family, and looking after elder members and infants is among the most important values.

Midwives expressed a generally positive opinion of the monitoring but had little direct interaction with the monitoring process. This may be in part attributable to the method of deploying the RM kit, which was very sensitive to the midwives' hierarchy in the community. The Mayan population is very keen to follow and respect traditions, and thus the role of the traditional midwife is instrumental in monitoring the pregnancies at the household. Thus, both doctors and nurses involved them at the monitoring sessions to assure better acceptance. Among the health personnel, responses indicated confidence in the value of the remote monitoring to identify risk situations. They supported incorporating the service as standard care.

The Mayan population has a profound respect for traditional values and interventions and therefore does not often use modern technology. Pregnant women and especially traditional midwives were reluctant at first to use a new technology. Nonetheless, many participants reported that the attractive design of the fetal monitor and the bonding between the nurses (who were fluent in Mayan) and the pregnant women eased its adoption.

### 3.3. Hardware and Software System Reports

Over the duration of the study, there were a total of 52 reported potential errors with the hardware and software systems. All potential errors were recorded by the field team in YUC and entered into a local copy of a spreadsheet to which all field team members had access (in some instances, reported errors were due to improper usage rather than hardware or software failure. For example, in one instance, the field team believed the prototypes were not performing properly. However, it was confirmed that the placement of the prototypes was incorrect, signaling the potential need for improved training with the system). Of the 52 reports, 24 were related to hardware, 15 to software, and 3 to connectivity, with an additional 10 classified as other.

All reports were reviewed within WHI's Quality Management System to ensure that the reports were evaluated and mitigated. Over the course of the study, the only reports that were not remedied were those related to physical deficits in the casing of the fetal monitor units. Ultimately, new cases were made to replace the deficient ones so that the study could continue. No physical deficiencies in the units were reported after replacement of the casing.

## 4. Discussion

The results of this study demonstrate both the feasibility and challenges of using technological interventions to improve MCH in rural regions. This study demonstrated that it is possible to achieve remote wireless antenatal fetal monitoring in a resource-poor and educationally challenged environment with a culturally diverse population. Results indicated a significant reduction in the number of participants requiring referral to the regional hospital for antepartum surveillance and an improved local adherence to antepartum fetal monitoring, as compared to hospital monitoring referral.

Given the restrictions of the small sample, the distribution of risk factors between intervention and control groups was not significantly different (see Table 2). As regards health outcomes, while there was an observed difference in the rate of eclampsia (3% in the control group versus 0% in the intervention arm) and gestational age (84% being 37+ weeks in the intervention arm versus 77% in the control group), neither of these differences achieved statistical significance. It is appreciated that one of the goals of antenatal monitoring (fetal heart rate, blood pressure, and urine) in the developed world is to diagnose preeclampsia and deliver the baby before the mother gets eclampsia. Therefore, this difference of eclampsia rate needs to be further explored and/or validated in future studies.

### 4.1. Adherence

In regard to appointment adherence, it is notable that women in the study group needed to travel comparatively short walking distances to the clinic, whereas individuals receiving standard of care had to travel up to 2 hours by car (if transportation was available) to the hospital and endure average waiting times that totaled 6–8 hours. These differences likely explain the difference in appointment adherence between those in the demonstration project and those receiving standard of care.

It is worth noting that, of the 40 women in the study group that were referred to the hospital, only 17 adhered. This lack of adherence, likely explicable by a combination of travel requirements and cultural challenges (hesitancy for women to travel alone), greatly limited the opportunity for impact of this screening methodology. It is important to emphasize that sociocultural factors need to be addressed along with the introduction of technology—for instance, financial support or childcare support for the woman's existing children provided on the day of appointment or having transportation provided may address such barriers.

### 4.2. Deployment Learning

The remote fetal monitoring demonstration project provided an opportunity to address barriers to enhance prenatal care. The program increased the workload and complexity for the Chemax clinic staff, requiring recruitment of additional nurses and the need for ongoing training.

Despite initial evidence of staff technical proficiency in system use, there were challenges with implementation. Previous to the study, physicians and nurses in the primary clinic had not directly cared for women with high-risk pregnancies, as previously these cases had been referred to the hospital for services. Project implementation resulted in a better-prepared first level of care team that was able to collect and transmit information appropriately to the specialist and directly deliver instructions back from the OB/GYN specialist to the participants. This enabled the specialist to identify which participants needed additional support. To successfully implement this, additional clinical training was required, including the execution and interpretation of a nonstress test.

Additionally, the project plan required adjustment to accommodate the Chemax clinic medical director's workload. Initially, the director was required to oversee use of the remote monitoring prototype technology kits and execute clinical directions provided by the hospital's OB/GYN specialist. However, the medical director's workload did not facilitate completion in a timely manner. Accordingly, the protocol was modified such that a dedicated primary care physician (PCP) was responsible for the remote monitoring session and supervision of the project nursing team.

There were further barriers associated with lack of human resources at the hospital level, as the two trained hospital OB/GYN specialists already had an excessive patient load. The project established workflow where the specialist would allocate time between 12:00 and 2:00 p.m. daily to review all daily monitoring sessions from the Chemax clinic. In this manner, the PCP could expect to have the monitoring sessions reviewed and commented on by the specialist in the early afternoon each day.

Additionally, all the medical data sources required validation, as the hospital and clinic used different medical record formats. These issues were addressed with the implementation of a simplified electronic record methodology for data capture and storage, with unified criteria for capturing the data. This required an initial internal training of the field team to coordinate all existing databases.

The PCPs demonstrated competence in identifying high-risk pregnancy factors and reasons for referral to the hospital but lacked knowledge regarding assessment of fetal monitor tests. None of the nurses were experienced with smartphone use (an integral component of the prototype technology kits), so there were significant educational challenges in recording and transmitting results. For example, nurses would often not upload all of the antepartum monitoring sessions, discarding sessions that they deemed of poor quality, because they felt such submission would affect their perceived performance. ICSS spent significant time training the nurses not only in performing fetal monitoring sessions but in using a smartphone for both simple tasks (opening an application, sending a text message) and more technical ones (reviewing cellular network coverage, substituting the battery, and changing a password). Overall, survey results demonstrated a significant acceptance of the monitoring at the clinic.

Due to lack of available staff, we were unable to immediately obtain all the required data related to home births performed by traditional midwives. Typically, these data are collected when the home-delivering mother subsequently comes to the clinic to obtain the birth certificate and vaccination schedule. Furthermore, we had to rely on scarce community resources (volunteers) to track and locate participants who did not show up for appointments.

### 4.3. Infrastructure Issues

The main infrastructure barriers included the unreliable electricity service, which impacted both the phone system and charging of prototype unit batteries. This ultimately required the addition of two electricity stabilizers. Cellular network coverage variations affected data upload and communication. The environmental conditions were hotter and more humid than expected, requiring the addition of air conditioning in the clinic fetal monitoring room.

In addition, there were infrastructure barriers at the hospital level. Despite recent hospital construction, the hospital did not have telephone lines, internet service, or computers, and the cellular network had intermittent coverage. ICSS provided computer equipment and mobile 3G/GSM SIM cards with sufficient data capacity, which not only facilitated data upload but also resolved the communication problems between the two distant healthcare units via the Web-based platform, phone calls, and/or text messages.

### 4.4. Sociocultural Challenges

Among the main sociocultural challenges experienced during the project were the native Mayan language and cultural barriers between the physicians and participants. Most women spoke Mayan and thus developed a closer relationship with the nurses, who were Mayan speakers, thus limiting direct communication with the PCP. One limitation of the interviews was the length of time between study informed consent, birth, and the administration of the questionnaires, which in some instances exceeded 4 months. It is also important to note that there are recognized strong Mayan cultural values focused on the umbilicus and the heart, which are associated with the perception of health balance. Furthermore, the overall number of participants queried was limited.

### 4.5. Technical Challenges

Study results indicated that new prototype technology may suffer hardware and software issues during implementation, so it is important to have adequate reporting tools as well as clear protocols and mitigation procedures in place to guarantee safe and effective execution. These results also highlighted the value of ongoing education and the importance of project oversight. Nonetheless, the hardware and software issues found during the course of the project did not impede the monitoring sessions.

Other technical challenges were related to clinic staff introducing unexpected protocol changes, none of which were part of the original study protocol. For example, nurses used several previously donated technologies during monitoring sessions, including a pencil-like Doppler monitor. Nurses used the Doppler monitor to prelocate the fetus within the mother's abdomen and then placed the prototype technology central unit at this location. However, the location site indicated by this pencil-like monitor did not correlate with the optimal site for the central unit, resulting in the perception of inferior fetal monitoring sensitivity from the prototype system.

Another deviation involved the use of a small pair of electrically powered audio speakers instead of the kit-provided headphones. Clinical staff introduced the speakers so that the nurses, mother, and family could all hear the baby's heart beat during the 30-minute recorded monitoring session. The audio speakers enabled a closer connection between the clinical staff, the mother, and her family; however, this deviation affected the ultrasound signal quality. This problem was identified after the nurses and field coordinator reported a decline in fetal monitoring system performance.

Software challenges included smartphones receiving text messages and calls during monitoring sessions, as well as participants installing applications not related to the study, which interfered with the clinical software application. Staff members were subsequently retrained on smartphone operation, and a single cell phone was assigned to the clinic for all phone communication. For this deployment, the system was used with a 3G cellular connection to upload the recorded data to the Internet where it was available to specialists in any of the remote sites. 3G coverage was intermittent, necessitating that sessions be stored in a system queue; the reception problems were more common during cloudy or rainy days or peak cell use hours. To remedy this situation, the project initiated a double login from the clinicians to assure the queued data would be appropriately uploaded once a proper signal was available.

The initial system design required each user to have a unique login ID and password. However, after several weeks of using the system, the clinical staff requested modification of the access case so all users could have the same login ID and password. The access was thus modified, but an unforeseen consequence occurred, resulting in looping of data uploads and duplicated copies of recordings. WHI engineers ultimately rewrote the software code to remedy this issue, and no impact on data occurred.

## 5. Conclusions

This study evaluated the feasibility and challenges of deploying a specially designed wireless, remote prototype technology system for antenatal fetal monitoring in a culturally diverse, resource-poor, and educationally challenged remote setting. Although there were many infrastructure, technical, and sociocultural challenges, this study nonetheless readily demonstrated that such prototype technology can be successfully adopted for use in a rural, resource-poor environment. Moreover, we observed more than twice as much adherence to antenatal monitoring for participants in the demonstration project as compared to the standard of care. However, differences in health outcomes were not observed, potentially due to cultural and logistical barriers that prevented women from accessing care at the hospital following diagnosis.

In regard to acceptability, pregnant participants, their husbands and/or partners, and midwives were receptive to the use of the prototype technology kits, as the study group maintained a high compliance of attendance to the Chemax clinic throughout the study. Both primary care staff and OB/GYN specialists supported the system's use and preferred it to the existing process of referrals to the hospital. The clinicians' rapid acceptance of the technology and their resourcefulness in following up with remote communications facilitated the study workflow. The technology facilitated the specialist being given advanced notification of any potentially concerning situation. However, as acknowledged above, the women did not always have readily available transportation for travel between the clinic and hospital, which may have limited their referral attendance.

In summary, this project demonstrates significant successes of an MCH intervention program and points to structural, educational, and cultural features that need to be addressed to assure more effective widespread dissemination in developing countries.

## Figures and Tables

**Table 1:** Salud Maya participant inclusion and exclusion criteria.

Inclusion (high-risk criteria)	Exclusion
<19 or >35 years old	<27 or >29 weeks of gestation

Three or more prior pregnancies	Twin pregnancy

Diabetes or gestational diabetes	Obstetric emergency or hemorrhage

Hypertension/preeclampsia or cardiomyopathy	Active labor

History of prior miscarriage	Epilepsy

Placenta previa	Dengue fever, influenza, or HIV

Kidney disease not requiring renal substitution therapy	Known allergies or hypersensitivities to plastics

Thyroid disease	History of a heart attack

**Table 2:** Distribution of risk factors^a^.

Risk factors	Study group	Standard of care
	(*n* = 74)	(*n* = 79)
<19 or >35	31%	37%
>3 pregnancies	68%	75%
Gestational diabetes	3%	3%
HBP/preeclampsia	18%	13%
Miscarriage	30%	27%
Placenta previa	3%	3%

^a^No statistically significant differences were found between the risk factors in the two groups per a binomial test with alpha = 0.05.

**Table 3:** Inclusion criteria for the selection of informant subjects for the qualitative surveys.

Stakeholders	Criteria
Clinical staff	Must be a primary care physician or nurse at the 1st level of care working in a facility that belongs to the ICSS AMANECE Valladolid program, treating pregnant women Must have approved the Virtual Diploma in Maternal and Child Health, granted by the National Polytechnic Institute and provided through ICSS' PIEENSO platform

Pregnant women	High-risk pregnant women being cared for at Chemax Health Services facility and monitored with the fetal monitoring kit Age: women 19 years of age and more, who represent different moments of life and thus have different views on their pregnancies: teenagers (19 to 20 years), young adults (21 to 34 years), and older adults (35 to 45 years) Educational level: literature indicates that education, particularly a woman's schooling, is a great contributor to her and her family's health Health insurance: people's insurance, IMSS, ISSSTE, or no insurance; this variable is linked directly with pregnant women's access to healthcare services and the type of services they receive (service portability) Special population group: for instance, indigenous populations have particular cultural aspects that are not seen in other population groups

Husbands/partners of pregnant women and midwives	Related persons to the pregnant women who have influence on the healthcare access decisions (partners, husbands, or midwives)

**Table 4:** Questionnaires for the informants (pregnant women and healthcare oersonnel).

Pregnant women: during pregnancy	Healthcare providers	Pregnant women: postpartum
Why did you accept the device?	Do you know how the device works, what is your opinion?	What do you think of the device that monitored your pregnancy?

How do you feel when you have the device on you?	Do you think the device is reliable?	Which do you think is more reliable: monitoring with the device or the directions from the midwife?

What have been some comments you have received from your relatives since you started using the device?	Do you think the operation of the device is complicated?	Did you trust the results of the device?

Have your expenses decreased with the use of the device?	Did you receive training prior to handling the device, how?	What do you think should improve the overall implementation of the device?

How much have you saved?	Have you had difficulties operating the device, which one?	What comments have you received from your family about the use of the prototype?

What do you do with the money you have saved?	How do you get organized to implement the device operation in pregnant patients?	Would you accept to use it again?

Do you understand the results that are shown in the device?	Did you observe any cultural inconvenience to use the device?	If it had any cost would you pay for it?

Would you recommend the use of this device to all pregnant women?	What have been the work flow changes in the process of caring for pregnant women during the consultation?	

Are there days that you have not come to receive the application of the device, why?	How do you think we can improve the implementation of this technology?	

What benefits do you think you get from this device?	List some basic features that you think are important to cover as health personnel that applies the device.	

Do you see any difficulty using the device?	Do you use some strategies to encourage participation from husband? Which?	

Would you use the device again in another pregnancy?		

**Table 5:** Questionnaires for husbands and midwives.

Husbands	Midwives
What do you think about the device your wife is using?	What have you heard of the device that is offered to pregnant women at risk?

Do you agree with this device that monitors the pregnancy of your wife? Why?	What do you think of the device?

What was your reaction when your wife first started using this device?	What do you think of the use of the device associated with the work you do?

How did you decide to use the device?	How often do pregnant women using the device visit you?

What did your wife tell you about the device?	Do you believe that pregnant women at risk feel safe and well cared for with the use of this device?

Have you seen any benefit in your economy with the use of the device?	What advantages have you observed from the device on your work as a midwife?

How often do you accompany your wife to the appointments?	What disadvantages does the use of the device bring to you?

What is your involvement during the consultation? Why?	Do you think there is any risk in using it?

What advantages do you think the device has?	Would you have liked to use the device?

What disadvantages do you think the device has?	Would you recommend the use of the device to all pregnant women?

	How is the care given to pregnant women at the clinic (nurses, physicians)?

**Table 6:** Information collected during regular monitoring sessions.

Baseline	Pregnancy	Postpartum
Obstetric information Medical record	Electronic database Medical record	Electronic database Medical record

Technical functionality for the remote monitoring kits		
Feedback form	Feedback form	Feedback form

Feasibility (study group); economic impact (study group)		
Pregnant women survey Husbands/partners survey Midwives survey Health personnel survey	Pregnant women survey Husbands/partners survey Midwives survey Health personnel survey	Pregnant women survey Husbands/partners survey Midwives survey Health personnel survey

**Table 7:** Place of delivery, obstetric complications, gestational age, and weight at birth^a^.

	Study group	Standard of care
	(*n* = 74)	(*n* = 79)
Place of delivery		
Hospital	74%	77%
At home/midwife	26%	23%

Obstetric complications		
Preeclampsia	11%	10%
Eclampsia	0%	3%
Hemorrhage	0%	0%
Sepsis	0%	0%

Gestational age at birth		
28 to 35 weeks	4%	3%
36 to 37 weeks	12%	20%
37+ weeks	84%	77%

Weight at birth		
Under 1000 gms	0%	0%
1000–1500 gms	0%	0%
1501–2500 gms	9%	6%
Over 2501 gms	91%	94%

^a^None of the differences between the two groups were statistically significant per a binomial test with alpha = 0.05.
